# Acute readmissions among care home residents aged 65+ years: a register-based study

**DOI:** 10.1007/s41999-025-01162-7

**Published:** 2025-02-21

**Authors:** Gitte Schultz Kristensen, Jens Søndergaard, Karen Andersen-Ranberg, Christian Backer Mogensen

**Affiliations:** 1 Emergency Department, Aabenraa Hospital, University Hospital of Southern Jutland, Aabenraa, Denmark; 2 Department of Regional Health Research, Faculty of Health Science, University of Southern Denmark, Odense, Denmark; 3 Head of research unit, Research Unit of General Practice, Department of Public Health, University of Southern Denmark, Odense, Denmark; 4 Department of Geriatric Medicine, Odense University Hospital and Head of Research Unit, Geriatric Research Unit, Department of Clinical Research, University of Southern Denmark, Odense, Denmark; 5 Department of Regional Health Research, Faculty of Health Science, University of Southern Denmark and Research Unit of Emergency Medicine, Aabenraa Hospital, University Hospital of Southern Denmark, Aabenraa, Denmark

**Keywords:** Care home, Readmission, Register-based, Risk factor

## Abstract

**Purpose:**

Care home residents are characterised by multimorbidity, cognitive impairment, and physical disabilities, resulting in a high risk of acute admissions and readmissions. The risk factors for acute readmissions may differ from those affecting older adults in the community. This study aims to identify risk factors associated with acute readmissions among care home residents within 30 days of discharge from an acute hospital admission.

**Methods:**

We included all care home residents aged 65 + years living in Southern Jutland in Denmark from 2014 to 2019 who were discharged from their first acute hospital admission (lasting 12 + h) in the study period. Data on baseline characteristics, index admissions, 30-day readmissions, and mortality were obtained from the highly valid Danish national health registries. Cox regression was employed to identify factors associated with acute readmissions.

**Results:**

The care home residents had a mean age of 83.9–86.2 at index admission, and 57.0–62.3% were women. Of 2108 initial admissions, 328 (15.6%) resulted in an acute readmission, and 302 (14.3%) died within 30 days of follow-up. Notably, nearly half of the readmissions occurred within 1 week of discharge. Being a new care home resident (care home residency < 3 months) was associated with acute readmission (HR 1.40), as was a medical history of cancer (HR 1.31), diabetes (HR 1.45), atrial fibrillation (HR 1.54), and COPD/asthma (HR 1.36). Conversely, dementia was associated with a significantly lower risk of acute readmission (HR 0.71).

**Conclusion:**

Our findings can help identify care home residents at elevated risk of readmission shortly after discharge.

**Supplementary Information:**

The online version contains supplementary material available at 10.1007/s41999-025-01162-7.

## Key summary points

**Aim:**

This study aims to identify risk factors associated with acute readmissions among care home residents within 30 days of discharge from an acute hospital admission.

**Findings:**

Being a new care home resident (care home residency < 3 months) was associated with acute readmission (HR 1.40), as was a medical history of cancer (HR 1.31), diabetes (HR 1.45), atrial fibrillation (HR 1.54), and COPD/asthma (HR 1.36). Conversely, dementia was associated with a significantly lower risk of acute readmission (HR 0.71).

**Message:**

Our findings can help identify care home residents at elevated risk of readmission shortly after discharge.

**Supplementary Information:**

The online version contains supplementary material available at 10.1007/s41999-025-01162-7.

## Background

Care home residents are particularly vulnerable due to a higher prevalence of cognitive impairment, physical disability, and multimorbidity compared to their community-dwelling peers [1, 2]. They face a high risk of experiencing acute hospital admissions, hospital adverse events, and acute readmissions [1, 3, 4]. The unique environment of care homes, featuring enhanced monitoring by healthcare workers, suggests that the factors and patterns driving readmissions among care home residents may differ from those affecting the general older population. Therefore, it is important to gain more knowledge about readmissions of care home residents to effectively target interventions designed to lower the risk of readmissions specifically from care homes.

Readmissions are often viewed as adverse events that significantly strain both patients and the healthcare system. In a recent systematic review [5], readmissions rates within 30 days for all adults aged 75 + years ranged from 10.3% to 37.6%. Moreover, readmission rates are higher among care home residents compared to the general population [5, 6], presumably due to increased frailty and multimorbidity.

Rates of readmissions are frequently used as indicators of the quality of hospital care [7]. While various interventions to prevent readmissions have been explored [8, 9], no single intervention has been proven to lower the risk of readmissions significantly [10]. However, multifaceted interventions involving both in- and outpatient settings and transitional care have successfully reduced readmission rates [11–15]. Identification of factors associated with early readmission is essential in the development, implementation, and execution of interventions designed to decrease the incidence of readmissions. Several studies have identified older age, male sex, multimorbidity, prolonged hospital stay, and care home residency as risk factors for readmission [5, 16, 17]. However, a literature search revealed only one study focusing specifically on risk factors of readmissions of care home residents: A retrospective cohort study from the United States [1]. Care home settings vary substantially across countries, and results from that study do not necessarily apply to Scandinavia [18].

A comprehensive understanding of readmissions among care home residents is crucial for ensuring improved care and finding the best medical treatment option for care home residents. The present register-based study aims to investigate acute readmissions among Danish care home residents within 30 days of discharge from an acute admission. Specifically, our objectives are to describe the resident's index admissions in terms of patterns for admissions and primary discharge diagnoses, estimate the incidence of acute readmissions among care home residents, time to readmission, and primary discharge diagnoses from readmissions, and to identify factors associated with acute readmissions among care home residents.

## Methods

### Study design, population and follow-up

#### Study design and population

**Fig. 1:**
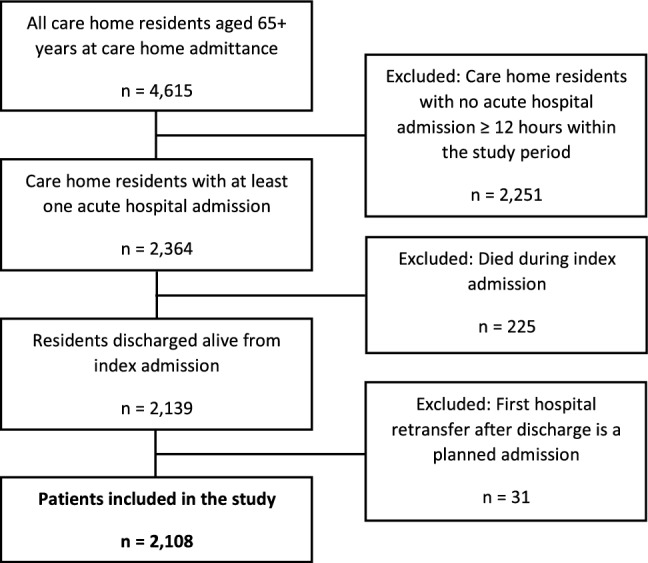
Flowchart of patients included in the study

This is a register-based population study. All care home residents living in Southern Jutland from 2014 to 2019 and aged 65 + years at care home admittance were eligible for inclusion. We included all residents discharged alive from any acute hospital admission (duration ≥ 12 h) between January 1, 2014, and December 31, 2019, regardless of diagnosis and discharge department (Emergency Department (ED) or in-hospital ward). Individuals not living permanently at a care home facility at the time of hospital discharge were excluded from the study. Patients with a planned hospital admission as the first hospital retransfer within 30 days of discharge were excluded. The in- and exclusion criteria are illustrated in Fig. 1. If a care home resident experienced multiple acute admissions during the study period, only the first admission was included as the index admission.

#### Follow-up and definition of readmissions

After discharge from the index admissions, patients were followed up for a maximum of 30 days until readmission, death, or the end of follow-up, whichever came first. An admission lasting at least 12 h in any Danish hospital within 30 days of discharge from index admission was classified as a readmission. Acute treat-and-release visits lasting less than 12 h were not classified as readmissions. Only the first readmission was included in the study.

### Setting

All Danish citizens are eligible to apply for a care home residency if they require extensive care due to permanent and substantial impairment of physical or mental function. Municipalities manage access to both public and private care home facilities, appointing residencies to citizens in greatest need, irrespective of financial means [19]. The municipalities govern all care homes and are subject to the Danish Law on Social Services. Care homes are staffed 24 h a day with nurse assistants and other healthcare professionals with 1.6–3.3 years of education, supplemented by unskilled workers and by nurses during daytime [20]. Community nurses, registered nurses employed by the municipalities and available for consultation 24 h a day, are on call during evenings, nights, and weekends. The community nurses handle dispensing medicine, wound care, and palliative care, among other tasks requiring nursing skills.

Southern Jutland is a geographical part of Denmark, comprising four municipalities with around 225,000 inhabitants in rural and urban areas. The population demographics in Southern Jutland are mainly similar to the Danish population; citizens aged 65 + years accounted for 23.4% of the population in Southern Jutland and 19.6% of the Danish population in 2019 [21]. Of all citizens aged 65 + years, care home residents accounted for 2.9% of the population in Southern Jutland and for 3.5% of the Danish population in 2019 [22]. The present study includes information from 2014 to 2019 on all residents of the 38 care homes in Southern Jutland, with approximately 1,600 long-term beds in total [23].

The Danish healthcare system is tax-funded and provides free and equal health care for all citizens [18]. Primary care physicians provide primary care and serve as gatekeepers to the secondary healthcare system, except in medical emergencies [24]. They also staff the out-of-hours service covering Southern Jutland. EDs operate 24/7, and nearly all patients are primarily seen in the ED, except for patients with prehospital identified cardiovascular disease or ongoing oncologic treatment. From the ED, patients are either discharged (treated and released within 12 h), admitted within the ED for expected short admissions (12–48 h), or transferred to an in-hospital ward for expected more prolonged admissions (> 48 h).

### Data collection

Data were collected from the following registries provided by the Danish Health Data Authority:

Care home residents were identified through the register-based Care Home Data (in Danish "Plejehjemsdata"), which contains valid data on all Danish citizens affiliated with a care home address from 2014 to the present [25, 26], including the date of moving to care home. Residents were identified with a civil registration number, a unique 10-digit identifier that links to other national registers [27].

Date of birth, date of death or migration (if relevant), and sex were collected from the Civil Registration System, which contains basic personal information on the entire Danish population since 1968 [27].

Data on in- and outpatient diagnoses, hospital admission dates, and outpatient visits was obtained from the Danish National Patient Register. The register contains information on all somatic inpatient admissions since 1977 and all in- and outpatient contacts to the health care system since 1995 (somatic and psychiatric) [28]. For every contact, one primary and optional secondary diagnoses are registered according to the International Classification of Diseases (ICD-10).

Information on filled prescriptions was obtained from the Danish National Prescription Registry, which contains individual-level data on all dispensing of prescription medicine since 1995 [29]. Medicines are categorised according to the Anatomical Therapeutic Chemical (ATC) classification system [30].

### Data variables

#### Baseline characteristics

We described patients in terms of sex, age, time since moving to the care home, and prevalence of morbidities at baseline, defined as the start date of the index admission. Data regarding morbidities were assessed by combining all primary and secondary ICD-10 diagnoses assigned to all in- and outpatient contacts 10 years before baseline with data on the use of prescription medicines during the past year from baseline. Patients were classified as having the morbidity of interest if they presented with a relevant ICD-10 code in hospital records and/or were users of prescription medicines indicated for that specific disease. To avoid overestimating the disease prevalence, the assessment did not include ATC codes if the particular medicine had several indications. The use of information from ICD-10 codes and ATC codes in assessing selected morbidities was developed for a previous study [31] and is described in Online Resource 1.

#### Index admissions characteristics

Index admissions were described in terms of duration of admission (12–48 h or > 48 h), length of stay, prior hospital contacts (including either a planned admission or an acute treat-and-release visit lasting less than 12 h within 30 days prior to the index admission), destination (discharged from the ED or ward), day and time of discharge, and diagnosis at discharge. Discharge diagnoses were assessed by using only the primary discharge diagnosis and grouped within the ICD-10 chapters.

#### Readmission characteristics

Readmissions were categorised as either short acute readmissions (12–48 h) or prolonged acute readmissions (> 48 h). Further descriptions of readmissions included days to readmission, the proportion of patients discharged from the same speciality and with the same diagnosis as in the index admissions, day and time of readmission, most frequent discharge diagnoses, in-hospital mortality, and 30-day mortality post-discharge.

### Data analysis and statistical methods

The distribution of care home residents regarding morbidities, sex, duration of index admission (12–48 h or > 48 h), hospital contacts before index admission, destination (discharged from ED or ward), day and time of discharge, and diagnosis at discharge, and all data on readmissions are presented as frequency counts and proportions. Age at index admission is presented as mean and standard deviation (SD). Length of stay in index admissions is presented as median with Interquartile Ranges (IQR).

Patients were grouped into three categories: Care home residents with no readmissions, with readmissions within 30 days of follow-up, and residents who died during the 30 days of follow-up without experiencing an acute hospital retransfer. All baseline characteristics and index admission characteristics were stratified by outcome category. We used Fisher's least significant difference procedure to test for differences between outcome groups. We used the Chi-Square Test for categorical variables and the Kruskal–Wallis test for continuous variables, with a significance level of *p* < 0.05.

#### Primary outcome: factors associated with readmission

Finally, we divided the cohort into two groups: Residents with and without readmissions within 30 days after discharge from index admission. The relation between specific baseline and index admission characteristics and readmission was analysed using Cox Regression, adjusting for competing risks by treating it as censoring. Readmission was the event of interest, while death due to any cause was the competing event preventing residents from experiencing the event of interest. Results are presented as fully adjusted cause-specific hazard ratios (HR) with 95% confidence intervals (CI). We adjusted for possible confounders based on clinical knowledge, as shown in Online Resource 2. The proportional hazards assumption was tested using the Schoenfeld residuals, and we found no violations.

### Ethics and approvals

The Danish Health Data Authority provided all data for the present study. No data were missing. The processing of personal data was approved by the Region of Southern Denmark (internal record 19/432,119) cf. Art 30 of The General Data Protection Regulation. According to Danish law, studies based solely on register data do not require approval from an ethics committee or informed consent from study participants [32].

## Results

Between January 1, 2014, and December 31, 2019, a total of 4,962 individuals resided in care homes in Southern Jutland, of which 4,615 were aged 65+ years at the time of moving to care homes (Fig. 1). Of these residents, 2,364 (51.2%) individuals experienced at least one acute hospital admission (duration ≥ 12 h), and 2,139 were discharged alive from their first acute admission during the study period. After excluding 31 residents with planned admission as their first hospital retransfer within 30 days of discharge, the final study cohort comprised 2,108 individuals. During the follow-up period, 328 (15.6%) of these residents experienced an acute readmission, while 302 (14.3%) died before the end of follow-up without experiencing an acute readmission.

### Baseline characteristics

Patient characteristics are presented in Table 1. There were no significant differences in age or sex between residents with and without readmission. However, residents who were readmitted within 30 days of discharge had a significantly higher prevalence of diabetes, chronic obstructive pulmonary disease (COPD) or asthma, and atrial fibrillation, along with a lower prevalence of dementia compared to those without readmissions. Additionally, readmissions were more frequent among residents who had moved to care homes within the past 3 months.

**Table 1:** Baseline characteristics at index admission of 2,108 care home residents living in Southern Jutland in 2014–2019, stratified by each category: Residents with no readmissions, residents with 30-day readmission, and residents who died during the 30 days of follow-up

Baseline characteristics	No readmission *n* = 1478 (70.1%)	30-day Readmission *n* = 328 (15.6%)	Significance testing^a^	Death during 30-day follow-up *n* = 302 (14.3%)	Significance testing^b^
Age in years, mean (SD)	84.4 (± 7.3)	83.9 (± 7.3)	ns	86.2 (± 6.9)	*p* < 0.001
Females, n (%)	921 (62.3%)	187 (57.0%)	ns	178 (58.9%)	ns
Morbidities, n (%)					
Cancer	313 (21.2%)	89 (27.1%)	ns	69 (22.8%)	ns
Diabetes	290 (19.6%)	89 (27.1%)	*p* = 0.003	51 (16.9%)	ns
Dementia	691 (46.8%)	127 (38.7%)	*p* = 0.008	165 (54.6%)	*p* = 0.012
Parkinson's disease	89 (6.0%)	22 (6.7%)	ns	24 (7.9%)	ns
Alcohol abuse	86 (5.8%)	18 (5.5%)	ns	11 (3.6%)	ns
Hypertension	829 (56.1%)	204 (62.2%)	ns	160 (53.0%)	ns
Ischemic heart disease	326 (22.1%)	80 (24.4%)	ns	66 (21.9%)	ns
Heart failure	208 (14.1%)	55 (16.8%)	ns	46 (15.2%)	ns
Atrial fibrillation	361 (24.4%)	114 (34.8%)	*p* < 0.001	73 (24.2%)	ns
Stroke	415 (28.1%)	92 (28.0%)	ns	95 (31.5%)	ns
COPD/asthma	287 (19.4%)	83 (25.3%)	*p* = 0.017	47 (15.6%)	ns
Number of selected morbidities			ns		ns
0	91 (6.2%)	14 (4.3%)		16 (5.3%)	
1–2	655 (44.3%)	126 (38.4%)		133 (44.0%)	
3–4	550 (37.2%)	128 (39.0%)		118 (39.1%)	
5+	182 (12.3%)	60 (18.3%)		35 (11.6%)	
Time since moving to care home, n (%)			*p* = 0.01		ns
0–90 days	366 (24.8%)	107 (32.6%)		80 (26.5%)	
91–365 days	421 (28.5%)	90 (27.4%)		72 (23.8%)	
365+ days	691 (46.8%)	131 (39.9%)		150 (49.7%)	

Ns, Not significant; COPD, Chronic Obstructive Pulmonary Disease

The percentages do not add up due to rounding

^a^Comparing baseline characteristics of care home residents without and with readmission within 30 days of discharge

^b^Comparing baseline characteristics of care home residents without readmission within 30 days of discharge to residents who died during the 30 days of follow-up

Residents who died during the 30 days of follow-up from the index admission were significantly older and had a higher prevalence of dementia while exhibiting an equivalent prevalence of the remaining morbidities presented in Table 1, compared to residents without readmissions.

### Index admission characteristics

Table 2 shows the characteristics of index admissions, comparing those with subsequent readmissions to those without and comparing admissions of residents who died after discharge to those without readmissions. Notably, admissions preceded by another hospital contact within 30 days (either a planned admission or an acute treat-and-release visit) were more likely to result in a readmission. There were no significant differences between admissions with and without subsequent readmissions regarding duration (12–48 or 48+ h), length of stay, ED discharge, weekend discharge, evening/night discharge, or discharge diagnosis.

**Table 2:** Index admission characteristics, stratified by each category: Residents with no readmissions, residents with 30-day readmission, and residents who died during the 30 days of follow-up

Admission characteristics	No readmission *n* = 1,478 (70.1%)	30-day Readmission *n* = 328 (15.6%)	Significance testing^b^	Death during 30-day follow-up *n* = 302 (14.3%)	Significance testing^c^
Duration 48 + hours	975 (66.0%)	218 (66.5%)	ns	223 (73.8%)	p = 0.008
Length of stay in days, median (IQR)	3 (1–7)	3 (1–7)	ns	5 (2–9)	p < 0.001
Previous hospital contact within 30 days^a^	106 (7.2%)	37 (11.3%)	p = 0.013	22 (7.3%)	ns
Discharged from ED	267 (18.1%)	63 (19.2%)	ns	35 (11.6%)	p = 0.006
Weekend discharge (Saturday–Sunday)	224 (15.2%)	51 (15.5%)	ns	32 (10.6%)	ns
Evening or night discharge (16.00–07.59)	279 (18.9%)	66 (20.1%)	ns	72 (23.8%)	ns
Most frequent primary discharge diagnoses			ns		ns
Pneumonia (J13-J18, J69)	176 (11.9%)	43 (13.1%)		47 (15.6%)	
Femur fractures (S72)	170 (11.5%)	30 (9.1%)		39 (12.9%)	
Urinary tract infection (N30, N390)	121 (8.2%)	22 (6.7%)		16 (5.3%)	
Other bacterial diseases (A3–A4)	84 (5.7%)	24 (7.3%)		19 (6.3%)	
Cognitive impairment (F00–F03, F05)	87 (5.9%)	17 (5.2%)		13 (4.3%)	
Cardiac diseases (I20–I25, I44–I49, I50)	67 (4.5%)	15 (4.6%)		12 (4.0%)	
Volume depletion or electrolyte disorders (E86–E87)	52 (3.5%)	22 (6.7%)		14 (4.6%)	
Symptoms, signs, and abnormal clinical and laboratory findings (R00–R99)	126 (8.5%)	23 (7.0%)		26 (8.6%)	
Other	595 (40.3%)	132 (40.2%)		116 (38.4%)	

Ns, Not significant

The percentages do not add up due to rounding

^a^A planned admission or an acute treat-and-release visit (< 12 h) terminated within 30 days before index admission

^b^Comparing baseline characteristics of care home residents without and with readmission within 30 days of discharge

^c^Comparing baseline characteristics of care home residents without readmission within 30 days of discharge to residents who died during the 30 days of follow-up

Residents who died during the follow-up period more often experienced prolonged index admissions, had more inpatient days and were less likely to be discharged from ED compared to those with no readmissions. They also tended to have a higher prevalence of pneumonia and femur fractures as the primary discharge diagnosis, although these results were not statistically significant.

A more detailed description of primary discharge diagnoses from index admissions is presented in Online Resource 3.

### Readmission characteristics

During the 30 days of follow-up, 328 (15.6%) patients were readmitted to hospital; of these, 31.4% required a short admission (12–48 h), while 68.6% required a more prolonged admission (> 48 h). Table 3 shows the admission characteristics of all readmissions. Nearly half of all readmissions occurred within the first week of discharge from index admissions (46.6%). Readmission was associated with high mortality rates, both in-hospital (10.7%) and 30 days post-discharge (20.1%).

**Table 3:** Characteristics of care home residents' readmissions within 30 days of discharge from acute admission

Readmissions *n* = 328	
Days from discharge to readmission	
0–1	53 (16.2%)
2–3	36 (11.0%)
4–7	64 (19.5%)
8–14	68 (20.7%)
15–30	107 (32.6%)
Re-attended to the same speciality	176 (53.7%)
Type of readmission	
Short admission (12–48 h)	103 (31.4%)
Longer admission (> 48 h)	225 (68.6%)
Day of readmission	
Weekday (Monday–Friday)	249 (75.9%)
Weekend (Saturday–Sunday)	79 (24.1%)
Time of readmission	
Dayshift (08.00–15.59)	184 (56.1%)
Evening (16.00–23.59)	111 (33.8%)
Night (00.00–07.59)	33 (10.1%)
Readmission discharge diagnosis^a^ same as at index admission	46 (14.0%)
In-hospital mortality	35 (10.7%)
Mortality 30 days post-discharge after readmission	66 (20.1%)

^a^Same groups of diagnoses as described in Table 2

Half (*n* = 176) of the readmitted residents returned to the same speciality from which they were discharged, and 46 (14.0%) were discharged from readmissions with the same diagnosis as in their index admission. The primary discharge diagnoses from readmissions were categorised into the same subgroups as those listed in Table 2.

When comparing the primary discharge diagnoses from index admissions and readmissions, we found a higher prevalence of pneumonia (16.2%), symptoms, signs, and abnormal clinical and laboratory findings (9.5%), and other bacterial diseases (8.8%) among readmitted patients. Conversely, there was a lower prevalence of femur fractures (4.0%) as the primary discharge diagnosis from readmissions. A more detailed description of the primary discharge diagnoses from readmissions is presented in Online Resource 4.

### Primary outcome: factors associated with readmissions

Table 4 lists the factors associated with acute readmissions. In the multivariate Cox proportional hazards model, the risk of acute readmissions increased significantly for residents with a medical history of cancer (HR 1.31), diabetes (HR 1.45), atrial fibrillation (HR 1.54), and COPD/asthma (HR 1.36), as well as for those with a higher number of morbidities. In contrast, dementia was the only morbidity associated with a significantly lower risk of acute readmission (HR 0.71).

**Table 4:** Baseline and index admission characteristics associated with acute readmissions among care home residents

	HR (95% CI) Fully adjusted analysis^a^
Baseline characteristics	
Age at index admission	0.99 (0.98–1.01)
Male sex	1.20 (0.97–1.49)
Morbidities	
Cancer	**1.31 (1.03–1.68)**
Diabetes	**1.45 (1.13–1.85)**
Dementia	**0.71 (0.57–0.89)**
Parkinson's disease	0.98 (0.63–1.52)
Alcohol abuse	0.83 (0.51–1.37)
Hypertension	1.20 (0.96–1.51)
Ischemic heart disease	1.04 (0.80–1.34)
Heart failure	1.10 (0.81–1.49)
Atrial fibrillation	**1.54 (1.22–1.95)**
Stroke	0.88 (0.69–1.13)
COPD/asthma	**1.36 (1.06–1.74)**
Number of selected morbidities	
0	1.00 (ref)
1–2	1.91 (0.89–4.10)
3–4	**2.17 (1.01–4.63)**
5 +	**2.64 (1.21–5.75)**
Time since care home admittance	
0–90 days	**1.40 (1.09–1.81)**
91–365 days	1.10 (0.84–1.44)
366 + days	1.00 (ref)
Admission characteristics	
Admission duration 48 + hours	0.93 (0.74–1.17)
Previous hospital contact within 30 days	**1.49 (1.06–2.10)**
Discharged from ED	1.18 (0.84–1.66)
Weekend discharge	1.05 (0.77–1.42)
Evening or night discharge	1.06 (0.81–1.39)
Discharge diagnosis	
Pneumonia (J13–J18, J69)	1.05 (0.74–1.49)
Femur fractures (S72)	0.83 (0.56–1.23)
Urinary tract infection (N30, N390)	0.85 (0.54–1.34)
Other bacterial diseases (A3–A4)	1.27 (0.82–1.96)
Cognitive impairment (F00–F03, F05)	0.89 (0.53–1.47)
Cardiac diseases (I20–I25, I44–I49, I50)	1.03 (0.61–1.77)
Volume depletion or electrolyte disorders (E86–E87)	**1.74 (1.11–2.74)**
Symptoms, signs, and abnormal clinical and laboratory findings (R00–R99)	0.83 (0.54–1.30)
Other	1.00 (ref)

COPD, Chronic obstructive pulmonary disease

Significant findings are in bold

^a^Adjusted for possible confounders concerning the given exposure based on clinical knowledge (see Online Resource 2)

Residents who recently moved to care homes had a higher risk of readmission (HR 1.40). Additionally, a previous hospital contact within 30 days of index admission (either a planned admission or an acute treat-and-release visit lasting less than 12 h) was associated with a higher readmission risk (HR 1.49). Among the primary discharge diagnosis groups from the index admission, only volume depletion or electrolyte disorders were associated with a higher risk of readmission (HR 1.74). Weekend or late discharges and the index admission length did not show a significant relationship with readmissions among care home residents.

## Discussion

To our knowledge, this is the first study investigating factors associated with acute readmissions of care home residents in Northern Europe. We provide register-based documentation on residents' index admissions and subsequent readmissions. Our findings indicate a high readmission rate of 15.6% and a significant out-of-hospital mortality 30 days post-discharge of 14.3%. Almost half of readmissions occurred within the first week following discharge, with pneumonia identified as the most frequent diagnosis at the time of readmission (16.2%).

We found that having another hospital contact within 30 days prior to the index admission—whether a planned admission or an acute treat-and-release visit lasting less than 12 h—was associated with an increased risk of subsequent readmission. Furthermore, individuals who had recently moved to care homes (within three months) exhibited a higher risk of readmission. This risk was also increased among residents with a higher number of morbidities or a medical history of cancer, diabetes, atrial fibrillation, or COPD/asthma. Conversely, a medical history of dementia was associated with a lower readmission risk.

Our study showed that 15.6% of care home residents discharged from acute hospital admissions had an acute readmission within 30 days, which is lower or equivalent to the reported rates of readmissions in studies on the community-dwelling geriatric population [5, 6]. The relatively low readmission rate may reflect differences in settings or study designs. However, it could also indicate that care home residency serves as a protective factor against readmissions. For instance, a Danish study on a transitional care intervention aimed at reducing 30-day readmissions reported 20–24% readmissions among community-dwelling older adults and only 16–19% among care home residents [15]. This lower readmission rate within the care home population may be attributed to improved surveillance, which allows for the treatment of acute illnesses within the care home itself, thereby preventing some hospital admissions. The presence of trained healthcare workers can facilitate earlier recognition of health deterioration, resulting in earlier initiation of treatment. Additionally, the lower readmission rate in the care home population might reflect advanced care planning, where decisions are made to avoid unnecessary hospital readmissions. This is further supported by our finding that 14.3% of care home residents died within 30 days after discharge from index admissions without experiencing a hospital retransfer.

Our primary outcome was to identify factors associated with acute readmissions. In our study, residents who had moved to care homes within the last three months showed a higher risk of readmission. A Danish study showed a substantial increase in hospital admissions nine months prior to care home admission, followed by a decline within 6 months after [33]. Markers of frailty, such as fatigue, malaise, and volume depletion, primarily drove the hospital admissions in relation to care home admission. Increased frailty at the time of moving into a care home likely contributes to a higher risk of both hospital admissions and readmissions. The increased risk of readmission among new care home residents may also reflect a lack of familiarity between residents and care home staff, which can delay end-of-life decision-making. Additionally, residents may feel insecure and unsettled in unfamiliar surroundings, potentially leading to delirium, falls or suspected infections. There should, therefore, be a focus on preventing readmissions among individuals who recently moved to care homes. This could include ensuring sufficient fluid intake, increasing focus on getting the care home resident and staff acquainted with one another, and arranging end-of-life conversations.

A hospital contact within 30 days prior to index admission—whether a planned admission or an acute treat-and-release visit—was associated with a greater risk of subsequent readmission. This may reflect increased morbidity and vulnerability following previous hospital contacts, which can impact index admissions and readmission risk. Studies on community-dwelling older adults have shown similar results [5, 6, 34]. Closer observation in the weeks following a hospital contact regarding signs of deterioration health may help prevent some repeat hospital visits.

Our study identified several morbidities associated with an increased risk of acute readmissions, including a medical history of cancer, diabetes, atrial fibrillation, and COPD/asthma. A recent systematic review on readmissions among community-dwelling older adults has shown similar results [5]. However, another systematic review has noted inconsistencies in baseline morbidities associated with readmissions in this population [6] and found that factors related to poor overall condition, such as immobility, chronic or acute confusion, and dependency in feeding, were stronger predictors of readmission. The study from the United States on risk factors for readmission among care home residents identified chronic kidney disease, pressure ulcers, and congestive heart failure as risk factors for early readmission [1]. These inconsistencies may reflect differences in settings and study designs. However, the inconsistencies also suggest that there may not be a strong association between individual morbidities and readmissions. Our findings indicate that the risk of readmission increases with a higher number of morbidities, highlighting the relationship between disease burden and vulnerability.

Interestingly, a medical history of dementia was associated with a significantly lower risk of readmission in our study, contrasting with findings from studies on community-dwelling older adults [5, 6, 35] and the United States study on care home residents [1]. A recent study from Northern Ireland supports our findings: Adults diagnosed with dementia showed a lower risk of 30-day readmission compared to the matched control group [36]. A Canadian study showed that severe cognitive impairment was associated with a lower risk of repeat ED visits [37]. Dementia as a protective factor against readmissions may reflect a practice of managing patients with cognitive impairment within the care home, thereby avoiding hospital retransfers when possible. However, dementia was also overrepresented among residents who died after discharge. Those who died without readmission were often older and had more prolonged index admissions. It is possible that some frail, older residents with advanced dementia were discharged to receive palliative care in the care home after prolonged hospital treatment without recovery, which may have contributed to the lower incidence of readmissions in this subgroup. This is supported by the United States study, where hospice or palliative care discharge plans were associated with a reduced risk of readmission [1], and by the study from Northern Ireland, where people with dementia had higher mortality after discharge from index admissions compared to the control group [36].

Contrary to studies on community-dwelling older adults [5, 6], our study did not find a relation between more extended in-hospital stays during index admission and an increased risk of readmission. This may be explained by a tendency to discharge care home residents as early as possible to avoid hospital adverse events, which could inadvertently increase the risk of readmission among those discharged early.

Volume depletion or electrolyte disorders were the only group of discharge diagnoses significantly associated with an increased readmission risk. This may result from an early discharge, where residents were not fully treated at the time of early discharge, resulting in readmission. Additionally, older adults with multimorbidity often present with non-specific complaints during acute illness, which makes it challenging to identify the underlying condition [38–40]. Dehydration and electrolyte disorders can indicate undetected illness, such as pneumonia or urinary tract infection, or may arise from adverse effects, e.g., diuretics. If underlying diseases are not adequately detected and treated sufficiently, this will naturally lead to an increased risk of readmission.

Almost half of the readmissions occurred within 7 days of discharge from index admissions, which aligns with findings from other Danish studies [16, 41]. This emphasises the necessity for interventions aimed at reducing readmissions from care homes to include immediate follow-up, e.g. by primary care physicians, because a planned follow-up consultation may be too late, even a week after discharge. Supporting this, a Danish study found that a follow-up visit by an outgoing multidisciplinary geriatric team within 1 week of discharge to a skilled nursing facility reduced 30-day readmission rates by 28%. The finding also indicates a need for close monitoring of residents for signs of deteriorating health in the first weeks after discharge.

Notably, two-fifths of readmissions occurred during evenings or nights, and nearly one in four took place on weekends, times when care homes typically lack nursing staff. In many of these cases, care home residents are assessed by visiting community nurses and subsequently referred to hospital by out-of-hours physicians. Therefore, interventions to reduce readmissions should also involve out-of-hours service and community nurses, as many residents experience urgent readmissions outside of regular primary care physicians' business hours.

A major strength of the present study is the complete cohort of all care home residents in Southern Jutland. The new Danish national care home register provides a reliable identification of true care home residents [26], significantly enhancing the quality of register-based studies on this population compared to earlier research [42]. Our study encompassed all residents with an acute index admission to any Danish hospital, including those outside Southern Jutland. This approach contrasts with studies based solely on individual hospital records, which often miss patients admitted to other facilities, potentially leading to a study population that does not accurately represent the broader demographic.

Moreover, studies that depend exclusively on hospital records risk overlooking readmissions to hospitals that are not included in their scope. By utilising register-based data, our methodology ensured complete follow-up for all patients, capturing all readmissions to any hospital as well as out-of-hospital mortality.

However, our study also has some limitations. Research based solely on registry data is constrained by information that may not be captured in the registries but is found in medical records. This includes morbidities that are not documented in hospital records or are never properly diagnosed, assessments of frailty, and clinical decisions such as "do not resuscitate" or "do not admit". Markers of frailty, such as immobility, dependency in feeding, and fatigue, may be among the strongest predictors of readmissions among care home residents but are not found in the hospital records. Furthermore, organisational aspects such as staff-to-resident ratios in care homes or educational level or personal experience among staff may also influence the risk of readmissions from care homes.

The present study focused exclusively on residents of care homes in Southern Jutland. Given that the Danish health registers cover the entire nation, a larger-scale study could reveal potential differences between municipalities or regions. As it is up to the 98 Danish municipalities to organise and manage the care homes as well as community nursing care, there can be differences in the patterns for readmission of care home residents between larger and smaller care homes or municipalities, rural and urban municipalities, etc.

The care home residents in this study are similar to those in other studies regarding age, sex, and prevalence of morbidities [43–45]. Their acute index admissions demonstrate comparable lengths of stay, primary discharge diagnoses, and post-discharge mortality rates compared to international studies [4, 46–48]. However, variations in healthcare systems and care home settings across countries may challenge the comparability of these results. We believe that our findings on factors associated with readmissions among care home residents are also relevant to other regions of Denmark and Scandinavia.

In this register-based study, the data did not allow us to identify the specific causes of readmissions. Some readmissions may result from the natural deterioration of one or more severe chronic diseases in residents who experience increased frailty after hospital discharge. Other readmissions may be more directly linked to index admissions, such as complications arising from treatment, hospital-acquired adverse events, or inappropriate transitions between hospital and care home settings. A more comprehensive assessment of medical records could reveal patterns or reasons for readmissions, leading to more targeted interventions or the development of predictive algorithms that would be beneficial for healthcare workers in Danish care homes. Qualitative studies could bring valuable insight into the perspectives of care home residents or staff, providing a more nuanced interpretation of the study results.

## Conclusion

In conclusion, this register-based study identified a high prevalence of early readmissions among care home residents. Factors associated with these readmissions included recent admission to care home, a previous hospital contact within 30 days, a higher number of morbidities, and a medical history of cancer, diabetes, atrial fibrillation, and COPD/asthma. Notably, a medical history of dementia was linked to a lower risk of readmissions.

## Supplementary Information

Below is the link to the electronic supplementary material.

Supplementary file1 (PDF 423 kb)

Supplementary file2 (PDF 419 kb)

Supplementary file3 (PDF 495 kb)

Supplementary file4 (PDF 476 kb)

## Data Availability

The data supporting this study's findings are available from the Danish Health Data Authority. Restrictions apply to the availability of these data, which were used under license for the current study and are not publicly available.

## Declarations

### Other

The preliminary results of the work were presented at the EuGMS conference in Valencia, Spain, in September 2024.
