# Supplementary material for: Acute readmissions among care home residents aged 65+ years: a register-based study
Source: Eur Geriatr Med. 2025 Feb 21;16(3):827–38. doi: 10.1007/s41999-025-01162-7 (PMC12174254; doi:10.1007/s41999-025-01162-7)
Supplement: Supplementary file 1 — Supplementary file1 (PDF 423 kb) [file 41999_2025_1162_MOESM1_ESM.pdf]

Title:

Acute readmissions among care home residents aged 65+ years – a register-based study

European Geriatric Medicine.

Authors

Gitte Schultz Kristensen*, MD, Emergency Department, Aabenraa Hospital, University Hospital of Southern Jutland. Department of Regional Health Research, Faculty of Health Science, University of Southern Denmark. ORCID id: 0000-0002-0238-5675

Jens Søndergaard, General Practitioner, Professor, Clin. Pharm., MD, Ph.D., Head of research unit, Research Unit of General Practice, Department of Public Health, University of Southern Denmark.

Karen Andersen-Ranberg, MD, Ph.D., Clinical Professor, Department of Geriatric Medicine, Odense University Hospital and Head of Research Unit, Geriatric Research Unit, Department of Clinical Research, University of Southern Denmark.

Christian Backer Mogensen, Consultant, Clinical Professor, MD, Ph.D., Department of Regional Health Research, Faculty of Health Science, the University of Southern Denmark and Research Unit of Emergency Medicine, Aabenraa Hospital, University Hospital of Southern Denmark.

*Corresponding author: gitte.schultz.kristensen@rsyd.dk

Online Resource 1: ICD-10 codes and ATC-codes used to define morbidities in Table 2.

**Comorbidity**	**IDC-10 codes given within the past ten years from baseline**	**ATC-codes of prescription medicines within the past year from baseline**
Cancer	C, D45-D47	L01
Diabetes	E10-E11, E13-E14	A10
Dementia	F00-F03, G30, G310B, G311, G318B, G318E, F1073, F1173, F1373, F1473, F1573, F1673, F1873, F1973	N06D
Parkinson's disease	F023, G20-G22	N04BA, N04BB, N04BD, N04BX
Alcohol abuse	F101-F109	N07BB
Hypertension	I10-I15	-
Ischemic heart disease	I20-I25	C01DA, C01DX16
Heart failure	I099A, I110, I130, I132, I420, I426-I427, I429, I50	-
Atrial fibrillation	I48	C01AA
Stroke	I60-I64, I69	-
COPD/asthma	J40-J47	R03, a minimum of two dispensed prescriptions within one year
