# Supplementary material for: Acute readmissions among care home residents aged 65+ years: a register-based study
Source: Eur Geriatr Med. 2025 Feb 21;16(3):827–38. doi: 10.1007/s41999-025-01162-7 (PMC12174254; doi:10.1007/s41999-025-01162-7)
Supplement: Supplementary file 2 — Supplementary file2 (PDF 419 kb) [file 41999_2025_1162_MOESM2_ESM.pdf]

Title:

Acute readmissions among care home residents aged 65+ years – a register-based study

European Geriatric Medicine.

Authors

Gitte Schultz Kristensen*, MD, Emergency Department, Aabenraa Hospital, University Hospital of Southern Jutland. Department of Regional Health Research, Faculty of Health Science, University of Southern Denmark. ORCID id: 0000-0002-0238-5675

Jens Søndergaard, General Practitioner, Professor, Clin. Pharm., MD, Ph.D., Head of research unit, Research Unit of General Practice, Department of Public Health, University of Southern Denmark.

Karen Andersen-Ranberg, MD, Ph.D., Clinical Professor, Department of Geriatric Medicine, Odense University Hospital and Head of Research Unit, Geriatric Research Unit, Department of Clinical Research, University of Southern Denmark.

Christian Backer Mogensen, Consultant, Clinical Professor, MD, Ph.D., Department of Regional Health Research, Faculty of Health Science, the University of Southern Denmark and Research Unit of Emergency Medicine, Aabenraa Hospital, University Hospital of Southern Denmark.

*Corresponding author: gitte.schultz.kristensen@rsyd.dk

Online Resource 2: List of possible confounders adjusted in relation to the given exposure in Table 6

**Exposure**	**Possible confounders**
Sex	-
Age at indexadmission	Sex
Cancer	Age, sex, alcohol abuse, diabetes, COPD/asthma
Diabetes	Age, sex, alcohol abuse
Dementia	Stroke, alcohol abuse, age, Parkinson's disease
Parkinsons' disease	Sex, age
Alcohol abuse	Sex, age
Hypertension	Sex, age, diabetes
Ischemic heart disease	Sex, age, diabetes, alcohol abuse, COPD/asthma
Heart failure	Ischemic heart disease, age, diabetes, hypertension, alcohol abuse
Atrial fibrillation	Age, hypertension, alcohol abuse, sex, ischemic heart disease
Stroke	Age, sex, atrial fibrillation, hypertension, COPD/asthma
COPD/asthma	Age, sex, alcohol abuse
Number of selected morbidities	Sex, age
Time since care home admittance	Sex, age
Admission duration 48+ hours	Age, sex, number of selected morbidities, previous hospital contact within 30 days
Previous hospital contacts within 30 days	Age, sex, time since care home admission, number of selected morbidities

Discharged from ED	Admission duration
Weekend discharge	Discharged from ED
Evening or night discharge	Discharged from ED
Discharge diagnoses	Age, sex
