# Supplementary material for: Acute readmissions among care home residents aged 65+ years: a register-based study
Source: Eur Geriatr Med. 2025 Feb 21;16(3):827–38. doi: 10.1007/s41999-025-01162-7 (PMC12174254; doi:10.1007/s41999-025-01162-7)
Supplement: Supplementary file 3 — Supplementary file3 (PDF 495 kb) [file 41999_2025_1162_MOESM3_ESM.pdf]

Title:

Acute readmissions among care home residents aged 65+ years – a register-based study

European Geriatric Medicine.

Authors

Gitte Schultz Kristensen*, MD, Emergency Department, Aabenraa Hospital, University Hospital of Southern Jutland. Department of Regional Health Research, Faculty of Health Science, University of Southern Denmark. ORCID id: 0000-0002-0238-5675

Jens Søndergaard, General Practitioner, Professor, Clin. Pharm., MD, Ph.D., Head of research unit, Research Unit of General Practice, Department of Public Health, University of Southern Denmark.

Karen Andersen-Ranberg, MD, Ph.D., Clinical Professor, Department of Geriatric Medicine, Odense University Hospital and Head of Research Unit, Geriatric Research Unit, Department of Clinical Research, University of Southern Denmark.

Christian Backer Mogensen, Consultant, Clinical Professor, MD, Ph.D., Department of Regional Health Research, Faculty of Health Science, the University of Southern Denmark and Research Unit of Emergency Medicine, Aabenraa Hospital, University Hospital of Southern Denmark.

*Corresponding author: gitte.schultz.kristensen@rsyd.dk

Online Resource 3: All primary discharge diagnoses from index admissions of care home residents in Southern Jutland in 2014-2019.

Chapter	Title	Subcategories	ICD-10 codes	Total N (%) Hereof
**I**	**Certain infectious and parasitic diseases**		**A00-B99**	**143 (6.8%)**
		Other bacterial diseases	A3-A4	127 (6.0%)
		Other		16 (0.8%)
**II**	**Neoplasms**		**C00-D48**	**15 (0.7%)**
**III**	**Diseases of the blood and blood-forming organs and certain disorders involving the immune mechanism**		**D50-D89**	**31 (1.5%)**
**IV**	**Endocrine, nutritional and metabolic diseases**		**E00-E90**	**104 (4.9%)**
		Diabetes mellitus	E10-E14	8 (0.4%)
		Volume depletion	E86	71 (3.4%)
		Other disorders of fluid, electrolyte and acid-base balance	E87	17 (0.8%)
		Other		8 (0.4%)
**V**	**Mental and behavioural disorders**		**F00-F99**	**156 (7.4%)**
		Dementia	F00-F03	77 (3.7%)
		Delirium	F05	40 (1.9%)
		Other		39 (1.9%)

**VI**	**Diseases of the nervous system**		**G00-G99**	**41 (1.9%)**
		Epilepsy and recurrent seizures	G40-G41	15 (0.7%)
		Other		26 (1.2%)
**IX**	**Diseases of the circulatory system**		**I00-I99**	**219 (10.4%)**
		Ischemic heart disease	I20-I25	20 (0.9%)
		Pulmonary embolism	I26	21 (1.0%)
		Conduction disorders	I44-I49	41 (1.9%)
		Heart failure	I50	33 (1.6%)
		Cerebrovascular diseases	I60-I69	57 (2.7%)
		Other		47 (2.2%)
**X**	**Diseases of the respiratory system**		**J00-J99**	**350 (16.6%)**
		Pneumonia	J13-J18	244 (11.6%)
		Chronic lower respiratory diseases	J40-J47	49 (2.3%)
		Pneumonitis due to solids and liquids	J69	22 (1.0%)
		Respiratory failure, not elsewhere classified	J96	14 (0.7%)
		Other		21 (1.0%)
**XI**	**Diseases of the digestive system**		**K00-K93**	**143 (6.8%)**
		Diseases of oesophagus, stomach and duodenum	K20-K31	35 (1.7%)
		Other diseases of intestines	K55-K64	47 (2.2%)
		Disorders of gallbladder, biliary tract and pancreas	K80-K87	28 (1.3%)
		Other		33 (1.6%)
**XIII**	**Diseases of the musculoskeletal system and connective tissue**		**M00-M99**	**21 (1.0%)**
**XIV**	**Diseases of the genitourinary system**		**N00-N99**	**197 (9.3%)**
		Renal failure	N17-N19	14 (0.7%)
		Urinary tract infection	N30, N390	159 (7.5%)
		Other		24 (1.1%)
**XVIII**	**Symptoms, signs and abnormal clinical and laboratory findings, not elsewhere classified**		**R00-R99**	**175 (8.3%)**
		Symptoms involving circulatory and respiratory systems	R00-R09	42 (2.0%)
		Symptoms involving digestive system and abdomen	R10-R19	36 (1.7%)
		Symptoms involving nervous and musculoskeletal systems	R25-R29	13 (0.6%)
		Symptoms involving cognition, perception, emotional state and behaviour	R40-R46	14 (0.7%)
		General symptoms and signs	R50-R69	54 (2.6%)
		Other		16 (0.8%)
**XIX**	**Injury, poisoning and certain other consequences of external causes**		**S00-T98**	**389 (18.5%)**
		Femur fractures	S720-S729	239 (11.3%)
		Other fractures	S02, S098B, S12, S22, S32, S42, S52, S62, S820-S829, S92	59 (2.8%)
		Intracranial injury	S06	20 (0.9%)
		Other		71 (3.4%)
**XXI**	**Factors influencing health status and contact with health services**		**Z00-Z99**	**109 (5.2%)**
		Medical observation and evaluation for suspected diseases and conditions, ruled out.	Z03	75 (3.6%)

		Other		34 (1.6%)
**Chapters VII-VIII, XII, XV-XVII and XX**				**15 (0.7%)**
	Total			2,108 (100.0%)

The percentages do not add up due to rounding.
