# Supplementary material for: Acute readmissions among care home residents aged 65+ years: a register-based study
Source: Eur Geriatr Med. 2025 Feb 21;16(3):827–38. doi: 10.1007/s41999-025-01162-7 (PMC12174254; doi:10.1007/s41999-025-01162-7)
Supplement: Supplementary file 4 — Supplementary file4 (PDF 476 kb) [file 41999_2025_1162_MOESM4_ESM.pdf]

Title:

Acute readmissions among care home residents aged 65+ years – a register-based study

European Geriatric Medicine.

Authors

Gitte Schultz Kristensen*, MD, Emergency Department, Aabenraa Hospital, University Hospital of Southern Jutland. Department of Regional Health Research, Faculty of Health Science, University of Southern Denmark. ORCID id: 0000-0002-0238-5675

Jens Søndergaard, General Practitioner, Professor, Clin. Pharm., MD, Ph.D., Head of research unit, Research Unit of General Practice, Department of Public Health, University of Southern Denmark.

Karen Andersen-Ranberg, MD, Ph.D., Clinical Professor, Department of Geriatric Medicine, Odense University Hospital and Head of Research Unit, Geriatric Research Unit, Department of Clinical Research, University of Southern Denmark.

Christian Backer Mogensen, Consultant, Clinical Professor, MD, Ph.D., Department of Regional Health Research, Faculty of Health Science, the University of Southern Denmark and Research Unit of Emergency Medicine, Aabenraa Hospital, University Hospital of Southern Denmark.

*Corresponding author: gitte.schultz.kristensen@rsyd.dk

Online Resource 4: All primary discharge diagnoses from readmissions of care home residents in Southern Jutland in 2014-2019.

Chapter	Title	Subcategories	ICD-10 codes	Total N (%) Hereof
**I**	**Certain infectious and parasitic diseases**		**A00-B99**	**36 (11.0%)**
		Other bacterial diseases	A3-A4	29 (8.8%)
		Other		7 (2.1%)
**III**	**Diseases of the blood and blood-forming organs and certain disorders involving the immune mechanism**		**D50-D89**	**5 (1.5%)**
**IV**	**Endocrine, nutritional and metabolic diseases**		**E00-E90**	**21 (6.4%)**
		Other disorders of fluid, electrolyte and acid-base balance	E87	5 (1.5%)
		Other		16 (4.9%)
**V**	**Mental and behavioural disorders**		**F00-F99**	**24 (7.3%)**
		Dementia	F00-F03	11 (3.4%)
		Delirium	F05	7 (2.1%)
		Other		6 (1.8%)
**VI**	**Diseases of the nervous system**		**G00-G99**	**8 (2.4%)**
**IX**	**Diseases of the circulatory system**		**I00-I99**	**26 (7.9%)**
		Conduction disorders	I44-I49	6 (1.8%)

		Cerebrovascular diseases	I60-I69	8 (2.4%)
		Other		12 (3.7%)
**X**	**Diseases of the respiratory system**		**J00-J99**	**76 (23.2%)**
		Pneumonia	J13-J18	42 (12.8%)
		Chronic lower respiratory diseases	J40-J47	12 (3.7%)
		Pneumonitis due to solids and liquids	J69	11 (3.4%)
		Other		11 (3.4%)
**XI**	**Diseases of the digestive system**		**K00-K93**	**28 (8.5%)**
		Diseases of oesophagus, stomach and duodenum	K20-K31	7 (2.1%)
		Other diseases of intestines	K55-K64	13 (4.0%)
		Other		8 (2.4%)
**XIV**	**Diseases of the genitourinary system**		**N00-N99**	**21 (6.4%)**
**XVIII**	**Symptoms, signs and abnormal clinical and laboratory findings, not elsewhere classified**		**R00-R99**	**31 (9.5%)**
		Symptoms involving circulatory and respiratory systems	R00-R09	9 (2.7%)
		Symptoms involving digestive system and abdomen	R10-R19	7 (2.1%)
		General symptoms and signs	R50-R69	8 (2.4%)
		Other		7 (2.1%)
**XIX**	**Injury, poisoning and certain other consequences of external causes**		**S00-T98**	**27 (8.2%)**
		Femur fractures	S720-S729	13 (4.0%)
		Other		14 (4.3%)
**XXI**	**Factors influencing health status and contact with health services**		**Z00-Z99**	**20 (6.1%)**
		Medical observation and evaluation for suspected diseases and conditions, ruled out.	Z03	10 (3.0%)
		Other		10 (3.0%)
**Chapters II, VII-VIII, XII-XIII, XV-XVII and XX**				**5 (1.5%)**
	Total			328 (100.0%)

The percentages do not add up due to rounding.
